# Poorly differentiated and anaplastic thyroid carcinomas: chromosomal and oligo-array profile of five new cell lines

**DOI:** 10.1038/sj.bjc.6603578

**Published:** 2007-04-03

**Authors:** R F Rodrigues, L Roque, T Krug, V Leite

**Affiliations:** 1 Cytogenetic Laboratory, Centro de Immunologia e Patologia Molecular, Portuguese Cancer Institute, R. Professor Lima Basto, Lisbon, Portugal; 2 Gene Express, Lda, Taguspark, Oeiras, Portugal; 3 Valeriano Leite: Molecular Endocrinology Laboratory, Centro de Immunologia e Patologia Molecular, Portuguese Cancer Institute, R. Professor Lima Basto, Lisbon, Portugal

**Keywords:** thyroid, CGH, expression, profile

## Abstract

Information on gene alterations associated to poorly differentiated (PDTC) and anaplastic thyroid carcinomas (ATC) is scarce. Using human cancer cell lines as a tool for gene discovery, we performed a cytogenetic and oligo-array analysis in five new cell lines derived from two PDTC and three ATC. In PDTC we evidenced, as important, the involvement of the MAPK/ERK kinase pathway, and downregulation of a group of suppressor genes that include E-cadherin. In ATC, downregulation of a specific group of oncosuppressor genes was also observed. Our ATC cell lines presented chromosomal markers of gene amplification, and we were able to identify for the first time the nature of the involved amplicon target genes. We found that the main molecular differences between the two cell line types were related to signal transduction pathways, cell adhesion and motility process. TaqMan experiments performed for five amplicon target genes and for two genes, which allowed a clear distinction between ATC and PDTC: *CDH13* and *PLAU* corroborated array results, not only in the cell lines, but also in an additional set of primary 14 PDTC and three ATC. We suggest that our findings may represent new tools for the development of more effective therapies to the hitherto untreatable ATC.

Thyroid malignant neoplasms of follicular cell origin are classified into four main categories: the differentiated papillary (PTC) and follicular (FTC) carcinomas, the poorly differentiated (PDTC) and the undifferentiated or anaplastic (ATC) carcinomas. The differentiated forms are the most common. Poorly differentiated thyroid carcinomas and ATC account for only 5–10% of the follicular malignancies; however, although rare, they represent a major challenge in oncology as they have a high morbidity and mortality rate (DeLelllis and Williams, 2004).

In recent years some progress has been obtained in the identification of the genomic changes associated to the pathogenesis of DTC. In PTC, it is now clear that, in a substantially fraction of tumours, malignant transformation takes place through the constitutive activation of effectors along the *MAPK/ERK* kinase pathway (Mellilo *et al*, 2005), and in FTC, the PAX8/PPRA*γ* fusion gene was determined to be an important event in the development of a subset of these carcinomas (Lacroix *et al*, 2005). At variance, the genetic information concerning PDTC and ATC is very limited. Only 27 PDTC have been evaluated by comparative genomic hybridisation (CGH) analysis, and as far as we are aware, there are no microarray studies on this entity (Wreesmann *et al*, 2002; Rodrigues *et al*, 2004). In ATC, it was demonstrated that *TP53* (Wynford-Thomas, 1997) and *β-*catenin (Garcia-Rostan *et al*, 1999) mutations as well as the overexpression of *OEATC1* (Mizutani *et al*, 2005) and *AURKB* (Sorrentino *et al*, 2005) were associated with ATC phenotype. These genes were demonstrated to be key effectors in anaplastic transformation, being responsible for evasion to apoptosis, enhanced tumour growth and replicative potential. However, although all efforts made, the molecular events related to their high invasion capacity, sustained angiogenesis and therapeutic resistance remain to be identified. By chromosomal studies it has become clear that these neoplasms were frequently characterised by cytogenetic markers of gene amplification: double minutes (dm) and homogeneously staining regions (hsr) (Roque *et al*, 1998). In other tumour types, such as neuroblastomas, the evaluation of these markers allowed the identification of specific oncogenes as targets of the amplification process, being found to be associated with a more aggressive biologic behaviour (Brodeur, 2003). In ATC, the identification of the amplified target genes has not been accomplished yet.

Cancer cell lines represent a pool of cells with an unlimited renewal and proliferative potential, and have proven to be invaluable in the research of aggressive rare tumours and a fundamental tool for gene discovery. The number of cell lines derived from poorly differentiated and ATC is small with just two PDTC cell lines (NPA and SMP) being referred in the literature (Asakawa *et al*, 1996; Ringel *et al*, 2000; Onda *et al*, 2004).

In our laboratory we were able to establish cell lines from two PDTC and three ATC, which we evaluated by conventional cytogenetics, CGH and oligonucleotide array analysis. It was our aim to characterise the cell lines at chromosomal and expression levels, providing information on the yet unknown deregulated genetic mechanisms associated to these two forms of aggressive thyroid neoplasias, namely to identify the target genes located at the amplicon units of the ATC cell lines.

From our results, it was evidenced that both cancer cell line types were characterised by largely altered chromosomal and expression profiles. In PDTC, we noted as significant the deregulation of MAPK pathway effectors and of a group of tumour suppressor genes. In ATC, we identified 40 genes as amplicon targets. Comparison of array results between the two types of cell lines revealed significant differences in the expression levels of genes related to signal transduction, cell adhesion, and motility. Array data were validated at RNA level through the performance of quantitative reverse transcription-polymerase chain reaction (RT–PCR) experiments for *ARNTL2, AURKA*, *CDH13, CLDN1*, *PLAU, PMAIP1*, and *RBAK.*

## MATERIALS AND METHODS

### Tumour specimens, conventional cytogenetics and CGH analysis

**Figure 1:**
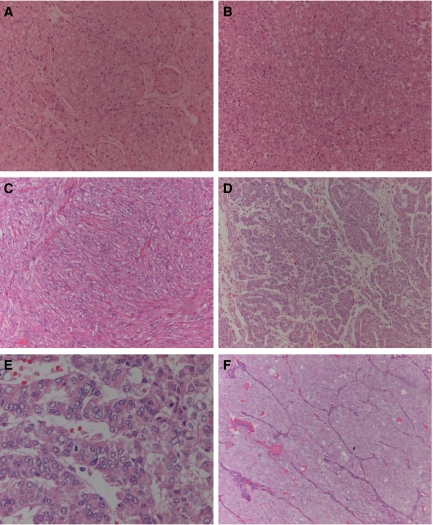
Representative haematoxylin–eosin-stained tissue sections of the primary poorly differentiated and anaplastic thyroid carcinomas from which the cell lines were derived. (**A**) T235 (original amplification 100); (**B**) T238 (original amplification 100); (**C**) T241 (original amplification 100); (**D**) T243 (original amplification 100); (**E**) T243 (original amplification 400); (**F**) T351 (original amplification 100).

Tissue samples were obtained by thyroidectomy from two patients with poorly differentiated papillary carcinomas and three patients with anaplastic carcinomas. Tumours were classified according to the criteria described in the Armed Forces Institute of Pathology (Rosai *et al*, 1992) and revaluated according to WHO histological classification of thyroid and parathyroid tumours by DeLellis and Williams (2004). Representative haematoxylin–eosin tissue sections from each of the primary tumours are shown in Figure 1A–F. A summary of the patient's clinical and histological data and the cell lines designation is depicted in Table 1.

Tumour cells were grown in Rosewell Park Memorial Institute 1640 (RPMI 1640) medium (Gibco-Invitrogen, Scotland, UK), containing 10% fetal calf serum (FCS) (Gibco-Invitrogen, Scotland, UK) and 1% antibiotic/antimicotic solution (Sigma-Aldrich Chemie, GmbH, Germany).

For conventional cytogenetic analysis cells were harvested after a 3- to 5-h treatment with colchicine (Karyomax-Gibco-Invitrogen, Scotland, UK). The metaphases obtained were GTG banded and karyotypes described according to International System for Human Cytogenetic Nomenclature (ISCN) (1995) (Mitelman, 1995).

Comparative genomic hybridisation of the five cell lines and evaluation of the obtained results were performed as described previously (Rodrigues *et al*, 2004).

### Oligonucleotide microarray analysis

Human thyroid total RNA from Clontech® (consisting in a pool of thyroid RNA obtained from 65 individuals who died from sudden death) was used as a normal baseline reference for microarray experiments. Total RNA from the cell lines was extracted using Trizol® (Gibco-Invitrogen, Scotland, UK), and cleaned up using Quiagen columns according to manufacturer's protocols. RNA integrity was assessed in the Bioanalyser Agilent 2100 and by visualisation of 28 and 18-s bands in agarose gel electrophoresis. Ribonucleic acid concentration was determined by spectrophotometry. For hybridisation the commercial *GeneChip*® *Human Genome U133 Plus 2.0 Array* from *Affymetrix*® were used.

For each cell line a pool of RNA derived from different passages was obtained, and samples were processed according to the experimental procedures specified by *Affymetrix*®.

The obtained microarrays were scanned in *GeneChip*® *Scanner* 3000 from *Affymetrix*®, controlled by a workstation with the *GeneChip*® *Operating Software* (GCOS) version 1.1.

### Statistical analysis

The software chosen for the analysis was the *DNA-Chip Analyser* (dChip) (copyright 2000–2004 Wang Lab, Harvard School of Public Health and Dana-Farber Cancer Institute). In the first step of the statistical analysis the arrays were normalised by the invariant set normalisation method, so that all non-biological variables were reduced, followed by a *model-based expression analysis*, using the model PM-only. The genes were filtered, so that those absent in all the samples were eliminated from the analysis. We considered genes to be differentially expressed between samples, when the lower bound of fold change (LBFC) was at least 2, with a confidence of 90%. In order to determine relevant genes overexpressed in the high-level amplification regions (HLA), we chose the set of genes that were found to have (i) an LBFC>5 in the comparison of the cell with the HLA to normal thyroid, within the HLA chromosomal region, and (ii) were commonly differentially expressed in the comparison of the three ATC cell lines to normal thyroid. The obtained data were then subject to hierarchical clustering and principal components analysis (PCA). The genes were also classified according to ‘gene ontology’ and ‘chromosome location’ using classify genes tool of D-Chip. We regarded only the *P* significant genes (*P*<0.001) for this classification.

### Quantitative real-time RT–PCR

To validate the array results, quantitative real-time RT–PCR was performed on seven genes: Aryl Hydrocarbon Receptor Nuclear Translocator-like 2 (*ARNTL2*); Aurora Kinase A (*AURKA*), Cadherin 13 (*CDH13*), Claudin 1 (*CLDN1*), phorbol-12-myristate-13-acetate-induced protein 1 (*PMAIP1*), plasminogen activator urokinase (*PLAU*), and RB-associated KRAB repressor (*RBAK*) using an ABI PRISM 7900HT Sequence Detection System (Applied Biosystems, Foster City, CA, USA). Sequence-specific primers and probes were selected from the Assay-on-Demand products (Applied Biosystems). As endogenous control we selected the housekeeping gene glyceraldehyde-3-phosphate dehydrogenase (GAPDH) from Applied Biosystems (HS:99999905_m1). The conditions of the TaqMan® PCR were as follows: 95°C for 10 min, followed by 40–45 cycles of 95°C for 15 s and 60°C for 1 min. The relative expression of each sample was calculated with respect to a standard calibration curve that represents a serial dilution of cDNA positive for the expression of the gene in analysis. Each sample was analysed three times and each PCR experiment included at least one non-template control well.

*ARNTL2, AURKA*, *CLDN1*, *PMAIP1*, and *RBAK* were chosen as representative overexpressed genes at the HLA regions. TaqMan® PCR experiments were performed in the three ATC cell lines reported herein as well as in three ATC independent primary tumours.

*CDH13* and *PLAU* were chosen because they were genes that accordingly to array data allowed a clear distinction between ATC and PDTC. Experiments were performed in the five cell lines described herein, in 14 PDTC primary tumours and in the previously aforementioned three ATC additional samples.

## RESULTS

### Conventional cytogenetic and CGH analysis

Tumours were characterised by karyotypes with both structural and numerical abnormalities (chromosomal supplementary data). Several populations were found in all cell lines . Different populations presented roughly the same alterations, differing only in ploidy. Maintenance of the karyotypes was observed for all cell lines.

**Figure 2:**
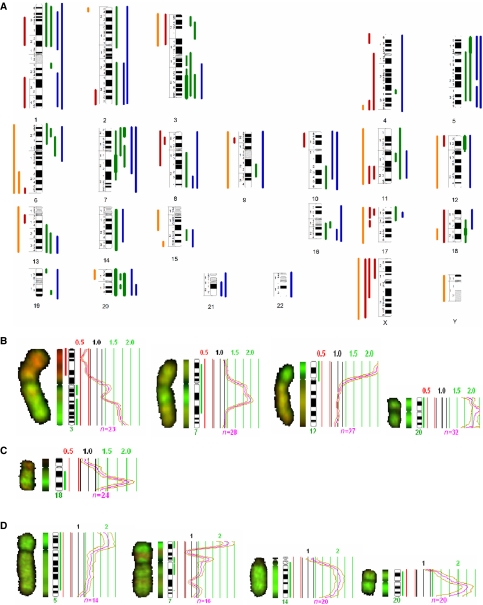
(**A**) Summary of the DNA copy number changes detected by CGH. Each bar in the left side of the chromosome ideograms represents a loss in one cell line (red—ATC, orange—PDTC) and each bar on the right side of represents a gain in one cell line (green—ATC, blue—PDTC). High-level amplifications are represented by thicker lines. (**B**) T235 HLA regions at 3q24-qter, 7q11.2-q22, 12pter-p11, and 20pter-qter; (**C**) T238 HLA at 18q21; (**D**) T241 HLA regions at 5pter-p12, 14q10-qter and 20p11.1-qter.

By CGH all cell lines presented multiple chromosomal imbalances. No significant modifications were observed throughout time at CGH level in any of the carcinomas after cell line establishment. A summary of the CGH imbalances is depicted in Figure 2A. Overall, gains of DNA sequences were more frequent than losses. High-level amplification regions were observed in the following cell lines: in T235 at regions 3q24-qter, 7q11.2-q22, 12pter-p11 and chromosome 20; in T238 at 18q21; and in T241 at 5pter-p12, 7pter-p21, 14q10-qter, and 20p11.2-qter (Figure 2B–D).

Comparative genomic hybridisation of the corresponding primary tumours have been performed and reported previously (Rodrigues *et al*, 2004). The chromosomal gains and losses observed for each of the cell lines and respective primary tumours were represented in an excel table (CGH supplementary data). Analysis of these data allowed to verify that although CGH alterations observed in the cell lines were not identical to the ones found in primary tumours, they presented major similarities, indicating that these cell lines largely reflect their primary tumour major cytogenetic imbalances.

Comparison of our PDTC and ATC CGH results with previously reported data (Hemmer *et al*, 1999; Wilkens *et al*, 2000; Wreesmann *et al*, 2002; Rodrigues *et al*, 2004) revealed that all imbalances, described as frequently occurring in the literature, were also observed in the cell lines.

### Oligonucleotide microarray analysis

For oligo-array analysis, only the cell lines were evaluated, owing to lack of well-preserved samples for RNA analysis.

Our work represents the first array expression profile in PDTC. Comparison of the gene expression profiles of the two PDTC cancer cell lines with normal thyroid uncovered 4238 significantly (*P*<0.001) differentially expressed genes. In all, 1079 genes were overexpressed and 3159 were underexpressed (array supplementary data). In Table 2 are depicted the 10 more overexpressed and the 10 more underexpressed genes in PDTC *vs* normal thyroid. After gene clustering of the differentially expressed genes according to their cellular function, the 10 main categories were in the order: ATP binding, cytoskeleton, cell cycle, mitosis, muscle development, DNA replication, tumour suppressor, transmembrane receptor protein tyrosine kinase signalling pathway, chromosome organisation and biogenesis, and RNA processing (array supplementary data).

When comparing gene expression profiles of the three ATC cell lines with normal thyroid, we found 3367 significantly (*P*<0.001) differentially expressed genes. In all, 1039 genes were overexpressed and 2328 were underexpressed (array supplementary data).

In Table 2 are depicted the 10 more overexpressed and underexpressed genes in ATC *vs* normal thyroid. In this group of tumours, gene clustering of the differentially expressed genes according to their cellular function revealed that the most representative were in the order: ATP binding, transport, extracellular space, cell growth and/or maintenance, cell adhesion molecule activity, cytoskeleton, soluble fraction, actin binding, extracellular matrix, and muscle development (array supplementary data). By array analysis we observed in ATC as well as in PDTC that expression downregulation was more relevant than upregulation, whereas by CGH gains were more frequent. A general lack of correlation between chromosomal CGH and array data has previously been described in other neoplasms, namely in colon cancer (Platzer *et al*, 2002), and may be explained by the recent observations demonstrating that changes in DNA copy number induce changes in the expression of only a limited number of genes. Moreover, the different sensitiveness of the two techniques must also be taken into account.

Comparison between the expression levels at the HLA regions in ATC and normal thyroid revealed that all the differentially expressed genes mapped at these chromosomal regions were overexpressed. In Table 3, we show the set of 40 genes identified to be commonly overexpressed in the three cell lines and mapping at the HLA regions. Of these, 37 represent known genes and three were ORFs (open reading frames).

When comparing the expression profiles of the two types of cell lines, we found 140 significantly differentially expressed genes (*P*<0.001). Of these, 33 were more expressed in PDTC cell lines and the other 107 were relatively more expressed in the ATC cell lines (array supplementary data). Clustering of these genes according to their cellular function revealed that the most representative group was that of signal transduction molecules followed by groups of genes involved in cell adhesion and motility process (array supplementary data).

**Figure 3:**
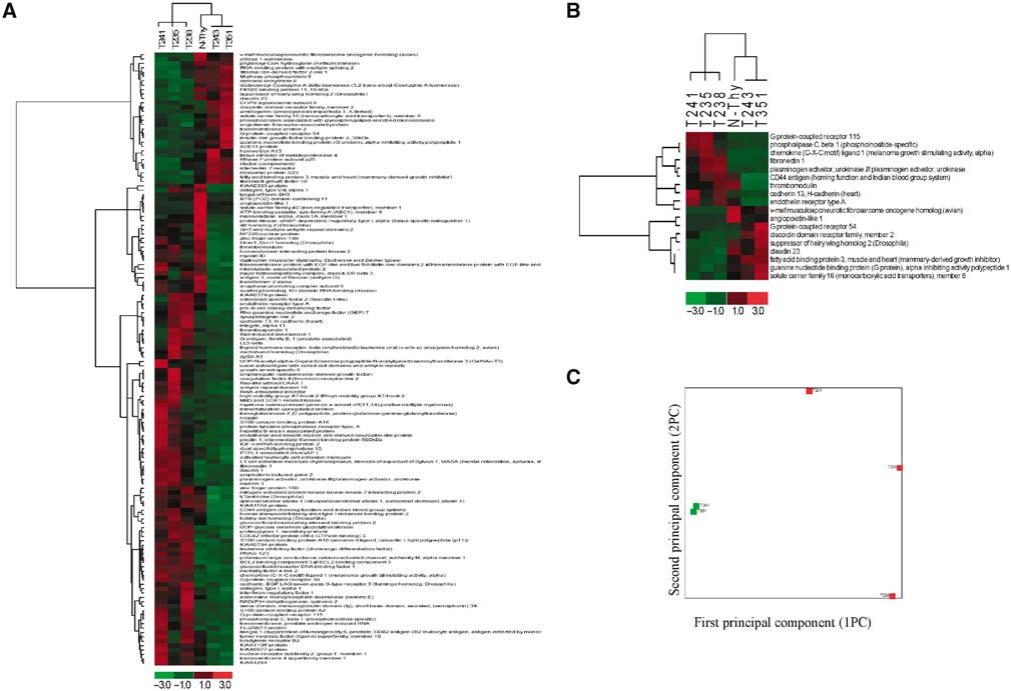
(**A**) Hierarchical clustering of the genes and samples using the 140 differentially expressed genes between the ATC and PDTC. Hierarchical clustering (**B**) and PCA analysis (**C**) of the genes and samples using the 18 genes that allowed a distinction between ATC and PDTC.

Hierarchical clustering using the 140 differentially expressed genes allowed a clear segregation of the tumour cells (Figure 3A). However, we could verify, when selecting only the nine more expressed genes in ATC and in PDTC, that a clear segregation of the two tumour types was still observable both my hierarchical clustering (Figure 3B) and PCA analysis (Figure 3C). All these molecules are known to be involved in cancer process, some being identified as tumour suppressor genes (e.g. *GPR54* and *CLDN23*) (Kotani *et al*, 2001; Katoh and Katoh, 2003) and others related to angiogenesis (e.g. *CXL1* and *EDNRA*) (Hosoda *et al*, 1992; Shintani *et al*, 2004) or cell migration capacities (e.g. *PLAU*) (Guo *et al*, 2002).

### Quantitative real-time PCR

**Figure 4:**
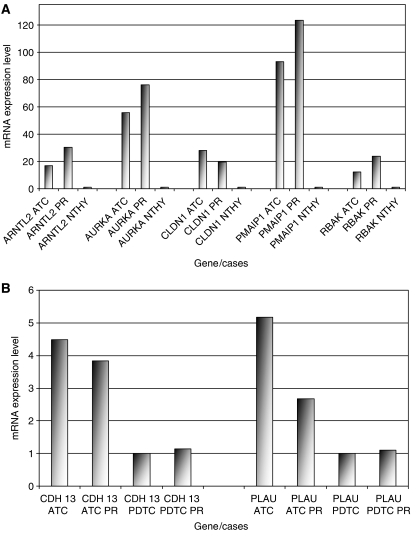
Real-time quantitative PCR/TaqMan results (measured by gene/GAPDH). The results were scaled by dividing each set of results by the minor value within the set. (**A**) Validation for five of the overexpressed genes within the amplicons, according to microarray analysis: *ARNTL2*, *AURKA*, *CLDN1*, *PMAIP1*, and *RBAK*. For each gene; first column—averages of the results obtained for all the analysed ATC cases (three cell lines+three primary cases); second column—averages for the three primary ATC; third column—results for normal thyroid. (**B**) Validation for two of the genes where expression was distinct in PDTC *vs* ATC, by array results: *CDH13* and *PLAU*. For each gene: first column—averages of the results obtained for all ATC analysed cases (three cell lines+three primary cases); second column—averages for the three primary ATC's; third column—averages of the results obtained for all PDTC analysed cases (two cell lines+14 primary cases); fourth column—averages for all the 14 primary PDTC.

Regarding the genes mapped at the HLA regions, TaqMan® PCR experiments confirmed the overexpression noted in microarrays analysis for all chosen genes: *ARNTL2, AURKA*, *CLDN1*, *PMAIP1*, and *RBAK.* Of remark is that the elevated expression values of these genes were also observed in the three additional ATC primary samples. As for *CDH13* and *PLAU*, TaqMan® PCR experiments also confirmed array results (Figure 4). Mann–Whitney statistical testing of the results revealed significant differences between the gene expression levels in the two types of neoplasms. The obtained *P*-values for *CDH13* was =0.0275 and for *PlAU* was =0.0377.

## DISCUSSION

Alterations in the structure or expression of proto-oncogenes and tumour suppressor genes were determined to be fundamental to cancer development and progression by respectively up- and downregulating cellular control pathways (Vogelstein and Kinzler, 1998). In thyroid the identification of these genes and how their alterations contribute to the pathogenesis of the two most aggressive cancer forms: poorly differentiated and anaplastic carcinomas are still ill defined. The use of cancer cell lines has represented an invaluable tool for gene discovery in other tumour types. As such, and to obtain information about the altered genes other than those already known to be associated to these two tumour types, we performed an evaluation at the DNA and RNA levels using cytogenetic and oligo-array analysis in five new cell lines established from two PDTC and three ATC.

Poorly differentiated thyroid carcinomas are classified according to the WHO as neoplasms presenting morphological and clinical criteria in between well-differentiated thyroid: papillary and follicular carcinomas, and anaplastic cancers.

Chromosomal translocations, inversions and duplications are the known mechanisms of oncogene activation in cancer. At the cytogenetic level, the most frequent structural rearrangements in papillary cancers are abnormalities with breakpoints at 10q11.2 (Roque *et al*, 2001). These alterations lead to the activation of the *RET* proto-oncogene. In our PDTC cell lines, although papillary features were still recognised, none of them presented alterations with breakpoints at 10q11.2 (*RET* gene was also observed not to be recombined – data not shown). However, of remark is the observation that by array analysis the most significantly overexpressed gene was *PBK*. The protein encoded by *PBK* is a serine/threonine kinase which belongs to the *MAPK* kinase protein family (Abe *et al*, 2000). The involvement of *PBK* has not previously been observed in thyroid cancer, but previous data (Mellilo *et al*, 2005) have demonstrated that the constitutive activation of effectors along the *MAPK/ERK* signalling pathway display a key role in the genesis and progression of a substantial proportion of papillary tumours. Our array findings thus reinforce the importance of this signalling pathway in the tumourigenesis of thyroid carcinomas with papillary characteristics.

*P53* is the only tumour suppressor gene identified to be involved in the pathogenesis of PDTC. Molecular studies revealed, however, that the frequency of tumours with mutations at this gene is low: ∼25% of cases (Wynford-Thomas, 1997). In our study, we were able to identify a group of other tumour suppressor genes which were downregulated in these carcinomas, and may have a key role in their development or progression. *PLAGL1* gene, which maps at 6q24.2, and *CDH1* mapping at 16q22.1, were those with the highest LBFC, respectively, −33.87 and –7.06. The involvement of *PLAGL1* has not been previously noted in PDTC. At variance, participation of *CDH1,* which codes for E-cadherin in thyroid tumorigenesis has been described. Rocha *et al* (2003) studied 17 cases of primary PDTC and showed that loss of E-cadherin was a common feature of these tumours.

In our array analysis, in the list of the 10 more underexpressed genes in ATC, we found thyroglobulin and thyroid peroxidase. This finding is in keeping with a preceding array study by Onda *et al* (2004) in a group of 11 ATC cell lines, and confirms previous studies demonstrating that in anaplastic tumours there is loss of the most characteristic thyroid cellular function, that is, synthesis of T4 and T3 hormones (Elisei *et al*, 1994). Known tumour suppressor genes, although not included in this list, were noted as downregulated in the array analysis for example, the *FHIT* (LBFC=−5.39) at 3p14.2; *ST7* (LBFC=−2.95) at 7q31 or *CLDN23* (LBFC=−4.58) at 8p23.1. Our results strengthen LOH studies (Kitamura *et al*, 2001) carried out in ATC that showed frequent allele loss at multiple chromosomal sites in this carcinoma type. Specifically interesting is the underexpression of the *ST7* tumour suppressor gene (Zenklusen *et al*, 2001) at 7q31, a region that has been reported as frequently deleted in ATC also by previous CGH studies (Rodrigues *et al*, 2004).

Gene amplification was observed to be a common phenomenon in ATC (Roque *et al*, 1998). In this study we were able to define for the first time the nature of the amplicon target genes present in this type of carcinomas. We found a set of 40 commonly overexpressed genes at the HLA (Table 3), which included molecules that are known to be involved in the regulation of centrosome duplication, chromosome orientation, and segregation process in cells such as: *AURKA*, *TPX2*, *CSE-1*, *MCM8* or *SMC4* (Adams *et al*, 2001). Genes related to transcription regulation (e.g. *RBAK* at 7p22.1 and *ARNTL* at 12p) and to cell adhesion process like *CLDN1.* Noteworthy was the observation of *PMAIP1* as an amplicon target at 18q21.32. *PMAIP1* is a gene expressed in cells in response to hypoxia, allowing cell adaptation to the altered metabolic demands induced by decreased oxygen levels (Yakovlev *et al*, 2004). Kim *et al* (2004) demonstrated that *PMAIP1* promoter directly responds to hypoxia via the hypoxia-induced factor (*HIF1A*). The *β*-subunit of *HIF1A* is *ARNT*. Of remark is the fact that *ARNTL2* was one of the overexpressed genes located at 12p11.3. Anaplastic thyroid carcinomas invade, infiltrate and rapidly grow in the surrounding tissues. In this context, we suggest that during the amplification process autocrine lopps are created that may confer important growth advantages to the neoplasms. The results obtained by TaqMan® PCR for *PMAIP1* and *ARNTL2*, but also for *AURKA*, *CLDN1*, and *RBAK* demonstrated that the amplification process of these genes is not only cell line specific, but also occurs in primary ATC. Therefore reinforcing the importance of these molecules in thyroid anaplastic characteristics.

Anaplastic thyroid carcinomas and PDTC have distinct disease courses. The former have a dismal prognosis, whereas in PDTC the median survival rate is 3–5 years (DeLellis and Williams, 2004). Assuming that these biologic different behaviours are a reflection of the deregulation of distinct molecular characteristics in each tumour type, we addressed this hypothesis by comparing the ATC and PDTC cell line expression profiles. We found that the main molecular differences between them were related to signal transduction pathways, cell adhesion and motility process, and this involved 140 genes.

Major advances were made in the treatment of chronic myeloid leukaemias and gastrointestinal tumours by using drugs that directly inhibit the pathways deregulated by the BCL2-ABL fusion gene and c-KIT mutations (Hochhaus, 2004). These genetic alterations are usually the only aberrations in these neoplasms at the time of diagnosis and treatment is in fact most of the times effective. Neoplasias such as PDTC or ATC are at variance characterised by a profound genomic instability and the currently used therapies especially in ATC cases are useless. In our work, hierarchical and PCA analysis allowed to verify that a group of 18 cancer genes served as a basis to differentially characterise anaplastic and poorly differentiated thyroid carcinomas. TaqMan® PCR experiments performed for two of these molecules, *CDH13* and *PLAU*, corroborated array results revealing significant expression levels differences between the two cancer types, not only in the cell lines, but also in an additional set of primary 14 PDTC and three ATC. Overall, our findings point out that the molecular pathways of *ARNTL2*, *AURKA*, *CDH13*, *CLDN1*, *PMAIP1*, *PLAU*, and *RBAK* should be further investigated order to understand the mechanisms by which these genes may be linked to anaplastic therapeutic resistance and to design concerted therapies that could improve ATC prognosis.

## Figures and Tables

**Table 1:** Clinical and histological data of the five cancer patients

**Case**	**Sex/age**	**Date of surgery**	**Deceased**	**Histologic diagnosis**
T235	F/77	12.05.97	17.06.97	Anaplastic thyroid carcinoma
T238	F/74	20.05.97	1.06.97	Anaplastic thyroid carcinoma
T241	F/72	30.06.97	30.11.97	Anaplastic thyroid carcinoma
T243	M/67	20.07.97	—	Poorly differentiated papillary thyroid carcinoma
T351	M/58	10.01.00	20.10.02	Poorly differentiated papillary thyroid carcinoma

**Table 2:** List of the 10 more up- and downregulated genes in the comparisons PDTC/normal thyroid and ATC/normal thyroid

**Gene**	**LBFC**
*The 10 genes more upregulated in the comparison PDTC/normal thyroid*	
T-LAK cell-originated protein kinase (*PBK*)	66.95
Prostate differentiation factor (*GDF15*)	43.56
Anillin, actin-binding protein (scraps homolog, *Drosophila*) (*ANLN*)	39.80
Leucine zipper protein FKSG14 (*FKSG14*)	31.82
Hyaluronan-mediated motility receptor (RHAMM) (*HMMR*)	28.71
Activin *β* E (*INHBE*)	27.29
Discs, large homolog 7 (*Drosophila*) (*DLG7*)	24.57
Thymidylate synthetase (*TYMS*)	24.14
Topoisomerase (DNA) II *α* 170 kDa (*TOP2A*)	23.84
Asp (abnormal spindle)-like, microcephaly associated (*Drosophila*) (*ASPM*)	23.64
	
*The 10 genes more downregulated in the comparison PDTC/normal thyroid*	
Lumican (*LUM*)	−233.11
Mucin 7, salivary (*MUC7*)	−152
Solute carrier family 26, member 4 (*SLC26A4*)	−133.11
Histatin 3 (*HTN3*)	−132.01
Histatin 1 (*HTN1*)	−122.6
Integral membrane protein 2A (*ITM2A*)	−107.75
Statherin (*STATH*)	−98.39
Amylase, *α* 2A; pancreatic (*AMY2A*)	−93
S100 calcium-binding protein A8 (calgranulin A) (*S100A8*)	−88.3
*Homo sapiens*-transcribed sequence with weak similarity to protein ref:NP_060312.1 (*H. sapiens*) hypothetical protein FLJ20489 [*Homo sapiens*]	−79.35
	
*The 10 genes more upregulated in the comparison ATC/normal thyroid*	
*Homo sapiens*-transcribed sequences	54.26
Phorbol-12-myristate-13-acetate-induced protein 1 (*PMAIP1*)	46.41
Fibronectin 1 (*FN1*)	41.06
Melanoma antigen, family A, 6 (*MAGEA6*)	31.36
L1 cell adhesion molecule (hydrocephalus, stenosis of aqueduct of Sylvius 1, MASA (mental retardation, aphasia, shuffling gait and adducted thumbs) syndrome, spastic paraplegia 1) (*L1CAM*)	30.98
Gb:AF043337.1 /DB_XREF=gi:12641914 /GEN=IL8 / FEA=FLmRNA /CNT=1 /TID=Hs.624.1 /TIER=FL /STK=0 /UG=Hs.624 /LL=3576 /DEF=Homo sapiens interleukin 8 C-terminal variant (IL8) mRNA, complete cds. / PROD=interleukin 8 C-terminal variant /FL=gb:AF043337.1	30.82
E2F transcription factor 7 (*E2F7*)	22.83
Human full-length cDNA 5′-end of clone CS0DK007YB08 of HeLa cells of *Homo sapiens* (human)	20.93
Melanoma antigen, family A, 3 (*MAGEA3*)	19.99
Anillin, actin-binding protein (scraps homolog, *Drosophila*) (*ANLN*)	19.74
	
*The 10 genes more downregulated in the comparison ATC/normal thyroid*	
Histatin 3 (*HTN3*)	−380.67
Solute carrier family 26, member 7 (*SLC26A7*)	−253.51
Histatin 1 (*HTN1*)	−198.24
Mucin 7, salivary (*MUC7*)	−177.54
Glycine amidinotransferase (L-arginine:glycine amidinotransferase) (*GATM*)	−159.71
Solute carrier family 26, member 4 (*SLC26A4*)	−149.79
Immunoglobulin J polypeptide, linker protein for immunoglobulin *α* and μ polypeptides (*IGJ*)	−127.34
Thyroglobulin (*TG*)	−118.95
Thyroid peroxidase (*TPO*)	−116.25
Integral membrane protein 2A (*ITM2A*)	−115.31

PDTC=poorly differentiated thyroid carcinomas.

ATC=anaplastic thyroid carcinomas.

LBFC=lower bound of fold change.

**Table 3:** Set of genes found to be commonly differentially expressed in the comparison of the three ATC cell lines to normal thyroid

**Genes**	**LBFC in 3ATC/NTHY**
#3*q*	
Claudin 1 (*CLDN1*)	19.09
Butyrylcholinesterase (*BCHE*)	8.59
SMC4 structural maintenance of chromosomes 4-like 1 (*SMC4L1*)	8.56
Epithelial cell transforming sequence 2 oncogene (*ECT2*)	6.85
Transferrin receptor (*p90, CD71*)	6.68
Karyopherin *α* 4 (importin *α* 3) (*KPNA4*)	4.56
BM-011 protein (*BM-011*)	3.18
Likely ortholog of mouse IRA1 protein (*IRA1*)	2.13
	
#*5*	
FLJ32363 protein (*FLJ32363*)	3.9
	
#*7*	
Activator of S-phase kinase (*ASK*)	8.44
Zinc finger protein 92 (*HTF12*)	8.01
FLJ20073 protein (*FLJ20073*)	7.54
Asparagine synthetase (*ASNS*)	6.91
RB-associated KRAB repressor (*RBAK*)	4.07
Hypothetical protein FLJ20097 (*FLJ20097*)	3.11
Origin recognition complex, subunit 5-like (yeast) (*ORCL5*)	2.45
Paternally expressed 10 (*PEG10*)	2.26
	
#*12*	
*RAD51*-interacting protein (*RAD51AP1*)	5.04
Forkhead box M1 (*FOXM1*)	3.63
Aryl hydrocarbon receptor nuclear translocator-like 2 (*ARNTL2*)	2.73
	
#*14*	
*KIAA1333* (*KIAA1333*)	3.29
Suppressor of Ty 16 homolog (*S. cerevisiae*) (*SUPT16 H*)	3.25
Mitogen-activated protein kinase kinase kinase kinase 5 (*ASK1*)	3.23
WD repeat and HMG-box DNA-binding protein 1 (*WDHD1*)	2.89
	
#18	
Phorbol-12-myristate-13-acetate-induced protein 1 (*PMAIP1*)	46.41
	
#*20*	
Ubiquitin-conjugating enzyme E2C (*UBEC2C*)	11.92
Synaptosomal-associated protein, 25 kDa (*SNAP25*)	7.14
Chromosome 20 open reading frame 97 (*C20ORF97*)	5.75
Aurora kinase A *(AURKA)*	5.62
Eukaryotic translation initiation factor 2, subunit 2 *β*, 38 kDa (*EIF2S2*)	3.98
Syntaxin 16 (*STX16*)	3.92
Chromosome 20 open reading frame 6 (*C20ORF6*)	3.77
CGI-09 protein (*CGI-09*)	3.72
TPX2, microtubule-associated protein homolog (*Xenopus laevis*) (*TPX2*)	3.56
Chromosome 20 open reading frame 100 (*C20ORF100*)	2.97
Phospholipase C, *β* 1 (phosphoinositide-specific) (*PLC beta*)	2.75
CSE1 chromosome segregation 1-like (yeast) (*CSE1*)	2.61
Solute carrier organic anion transporter family, member 4A1 (*SLCO4A1*)	2.58
Transglutaminase 2 (C polypeptide, protein-glutamine- *γ*-glutamyltransferase) (*TGM2*)	2.6
MCM8 minichromosome maintenance deficient 8 (*S. cerevisiae*) (*MCM8*)	2.22

ATC=anaplastic thyroid carcinomas; HLA=high-level amplification regions; LBFC=lower bound of fold change.
