# Supplementary material for: Poorly differentiated and anaplastic thyroid carcinomas: chromosomal and oligo-array profile of five new cell lines
Source: Br J Cancer. 2007 Apr 3;96(8):1237–45. doi: 10.1038/sj.bjc.6603578 (PMC2360140; doi:10.1038/sj.bjc.6603578)
Supplement: Supplementary Data 3 [file 6603578x3.doc]

**Chromosomal Supplementary Data** – ATC^*^ and PDTC^§^ cell lines: Karyotypes of the most representative populations and CGH^¥^ results

| Case | Karyotype | **CGH Results** |
| --- | --- | --- |
| T235 (ATC) | 73~79<69>XX[4], -1[2], add(1)(p?11)[1], der(1;9)(q10;q10)[2], + iso(1)(p)[1], -2[3], der(2)t(2;5)(q31~32;q21)[2], del(3)(p12~13p21)x2[4], der(3)t(2;3)(?;p21)t(2;3)(?;q27)[3], +der(3)t(2;3)(q22;p21)t(2;3)(?;q27)[4], +der(3)t(2;3)(?;?)t(2;3)(?;?)[3], +der(3)t(2;3)(?;?)t(3;16)(?;?)[4], der(4;14)(q10;q10)[3]x2[1], +del(5)(q10)[1], +der(5)t(5;11;22)(p15.1;?;?)[3], +iso(5)(q)[1], -6[3], -6[1], +7[4], +7[1], +del(7)(q31)[3], der(8;X)(q10;q10)[1]x2[3], der(9;17)(q10;q10)x2[4], der(10)t(10;10)(p11;q22)[2]x2[2], -11[2], +11[1], +12[1], +del(12)(q21)[1], +der(12)t(12;12)(p11;?)del(12)(q11q21)[3]x2[1], -13[1], +13[1], -14[3], -14[1], -15[1], add(15)(p11)[2], -16[1], +17[4], +17[1], -18[4], -19[1], +19[1], +20[4], +20[2], +20[1], +20[1], + iso(20)(p)[3], -21[4], -21[2], -21[1], -22[2], der(?;15)(?;q15)[1], +mar1~2[3][cp4] | -1p33-p21, -1q31-qter, +2pter-q11.2, +2q22-qter, -3pter- p14, +3q11.2-q13.31, +3q21.3-qter, -4q10-qter, +5p12-q33, +6pter-p21.1, +7p21-q31, -8p22-p21, +8q12-qter, -10pter-p12, +10q22.2-qter, +11q12-q13, -11q21-q23, +12pter-q11, +16q22-qter, -17p13.1-p12, -18, +19p13.3, +20, -Xpter-p11.2.  **HLA** at:** 3q24-qter, 7q11.2-q22, 12pter-p11, 20pter-qter. |
| T238 (ATC) | 50~55<46>X[5], -X[5], der(1;14)(p10;q10)[5], +der(1)t(1;1)(q12;p11)[5], +2[1], del(2)(q35) [1], +del(3)(p11p15)[5], +add(4)(p11)[1], +der(4)t(4;7)(q2?8;q?)[2],+5[4], +der (5;14)(p10;q10)[1], der(7)t(4;7)(q?;q22)[5], +8[1], -10[1], +10[1], +11[5], +14[4], -16[1], ?16[1], +der(18)t(18;18)hsr(18)(p11 ;?)t(X;18)(?;?)[5], -19[1], +der(19)t(13;19)(q?;q13)[4], +der(19)t(13;19)(q?;q13)t(13 ;19)(q?;q?)t(13;19)(q?;q?)[1], +20[4], +22[1], +mar1~3[3], +tr[1] [cp5] | +1pter-p21, +3p25-p24, +3p11-q28, +6p23-p22, -6q26-qter, +7pter-q11.2, +11pter-q23, +13q14.2-qter, +17pter-p11.2, +18q11-qter, +19q13.1, +20p12-p11.2, +20q11.2-q12, -Xpter-q21.  **HLA at:** 18q21. |
| T241 (ATC) | 42~52<46>X[4]X[2], -X[3], inv(X)(q24q13)[1], add(1)(p11)[1], del(1)(p13)[2], +del(1)(q10)[1], der(1;14)(p10;q10)[1], +der(1;21)(q10;q10)[1], -2[2], del(2)(q?33)[3], dic(2;6)(q37;q27)[1], der(3)ins(3;7)(q27;?)ins(3)(q27q24q26)[6], del(4)(p10)[1], der(5;15)(p10;q10)[4], +6[1], der(7)del(7)(p149del(7)(q11.2q22)dup(7)(q32q36)[6],  -8[6], add(8)(p12)[1], -9[1], -9[1], del(9)(p22)[3], der(9)t(9;15)(p22;q15)[3]x2[2], -11[1], add(11)(q14-21)[1], add(11)(q23-25)[2], -12[2], der(12;15)(q10;q10)[3]x2[2], -13[6],  -13[1], der(13)t(13;14)(p11;?)[5], +14[1], +der(14)t(3;14)(?;p11)[3], +der(14)t(13;14)(q?21;p11)[5], -15[1], -17[1], -18[1], -19[2], ?20[1], der(20;21)(q10;q10)[4]x2[1]x3[1], +der(20)t(9;20)(?,p12)x2[6], +iso(20)(p)[1], +21[2], iso(22)(q)[6], +mar1~2[2][cp6] | +1p36.2-p21, +1q21, -2q34-qter, +3p24.2-p21, +3q24-qter, -4pter-p15.1, +4q31, -4q33-qter, +5pter-q33, + 6p21-q12, +7pter-p21, +7p12-q21, -8pter-q21.2, -9p21, +9q21.3-q31, -11q22-qter, -12pter-p12, -13q12-q13, + 13q21.3-qter, +14q10-qter, +15q22-q24, +16q11.2-q13, -17p13-p11.2, -17q11.2-q21.1, +20p12-qter, -X.  **HLA at:** 5pter-p12, 7pter-p21, 14q10-qter, 20p11.2-qter |

**Table 2 (continued)**

| Case | Karyotype | **CGH Results** |
| --- | --- | --- |
| T243(PTCPDTC) | 60~73<69>, XY,-X[7],-Y[3], add(X)(q11)[4], del(X)(q11)[4], -1[3], del(1)(q11)[4], der(1)del(1)(p32-34)ins(1)(q42?q)dup(1)(q21-q42-qter)[4], -2[7], del(3)(p11)[3], -4[4], -5[3], add(5)(q11)(q11)[3], del(5)(q11)[3], add(6)(q11)[4], del(6)(q11)[6], +del(7)(q11)[5], -11[5], del(11)(q11)[2], add(12)(p11)[3], del(12)(p11)[5], -13[6], -13[4], -14[3], add(14)(p11)[2], -15[6], -16[6], +17[2], -18[6], +20[3], -21[4], -22[6], -22[4], +mar1-11[cp7] * | +1p35-p13, +1q24-q43, -3pter-p25, +3q22-qter, -4q35-qter, +5pter-q21,+ 5q31-qter, +6pter-q14, -6q22-qter, +7pter-q31.1, +8p23.1-qter, +9, +10pter-q24, +11q11-q23, +12, +13q22-qter, -15q26-qter, +16q22-q23, +17p12-p11.2, +20p13-qter, +21q11.2-q22, +22q11.2-q12, -X, -Y |
| T351(PTCPDTC) | 35~39<46>XY[17], der(3;20)(q10;q10)[18],-6[18],-9[18],-11[18],-13[18], der(14)t(14;15)(q32;q22)[18],-15[18],-17[18], del(18)(q21)[18] [cp18] | +1, +2, -3pter-p12.3, +3q13.3-qter, +4, +5, -6, +7, +8, -9, +10, -11, -12, -13, +14q10-q24, -15q10-q21, +15q22-qter, +16, -17, +18pter-q12, -18q21-q22, +19, -20p13-p11.2, +20q11.2-qter, +21, +22. |

^*^ATC – anaplastic thyroid carcinomas; ^§^PDTC – poorly differentiated thyroid carcinoma; ^¥^CGH – comparative genomic hybridization; ^**^HLA – high level amplification
